# A convolutional neural network for face mask detection in IoT-based smart healthcare systems

**DOI:** 10.3389/fphys.2023.1143249

**Published:** 2023-03-31

**Authors:** Bose S., Logeswari G., Thavavel Vaiyapuri, Tariq Ahamed Ahanger, Fadl Dahan, Fahima Hajjej, Ismail Keshta, Majed Alsafyani, Roobaea Alroobaea, Kaamran Raahemifar

**Affiliations:** ^1^ Department of Computer Science and Engineering, CEG Campus, Anna University, Chennai, Tamil Nadu, India; ^2^ Department of Computer Sciences, College of Computer engineering and sciences, Prince Sattam Bin AbdulAziz University, Al-Kharj, Saudi Arabia; ^3^ Department of Management Information Systems, College of Business Administration, Prince Sattam Bin Abdulaziz University, Al-Kharj, Saudi Arabia; ^4^ Department of Management Information Systems, College of Business Administration Hawtat Bani Tamim, Prince Sattam Bin Abdulaziz University, Al-Kharj, Saudi Arabia; ^5^ Department of Information Systems, College of Computer and Information Sciences, Princess Nourah bint Abdulrahman University, Riyadh, Saudi Arabia; ^6^ Computer Science and Information Systems Department, College of Applied Sciences, AlMaarefa University, Riyadh, Saudi Arabia; ^7^ Department of Computer Science, College of Computers and Information Technology, Taif University, Al-Hawiyya, Saudi Arabia; ^8^ College of Information Sciences and Technology, Data Science and Artificial Intelligence Program, State College, Penn State University, State College, PA, United States; ^9^ School of Optometry and Vision Science, Faculty of Science, University of Waterloo, Waterloo, ON, Canada; ^10^ Faculty of Engineering, University of Waterloo, Waterloo, ON, Canada

**Keywords:** COVID-19, image processing, neural network, face mask and social distancing detection, convolutio nal neural networks (CNN)

## Abstract

The new coronavirus that produced the pandemic known as COVID-19 has been going across the world for a while. Nearly every area of development has been impacted by COVID-19. There is an urgent need for improvement in the healthcare system. However, this contagious illness can be controlled by appropriately donning a facial mask. If people keep a strong social distance and wear face masks, COVID-19 can be controlled. A method for detecting these violations is proposed in this paper. These infractions include failing to wear a facemask and failing to maintain social distancing. To train a deep learning architecture, a dataset compiled from several sources is used. To compute the distance between two people in a particular area and also predicts the people wearing and not wearing the mask, The proposed system makes use of YOLOv3 architecture and computer vision. The goal of this research is to provide valuable tool for reducing the transmission of this contagious disease in various environments, including streets and supermarkets. The proposed system is evaluated using the COCO dataset. It is evident from the experimental analysis that the proposed system performs well in predicting the people wearing the mask because it has acquired an accuracy of 99.2% and an F1-score of 0.99.

## 1 Introduction

Middle East Respiratory Syndrome (MERS) and Severe Acute Respiratory Syndrome are two life-threatening illnesses that can be brought on by the virus class known as coronaviruses (CoV), which can also cause the common cold (SARS). Since the COVID-19 pandemic swept the globe, governments around the world have taken tough but necessary steps to stem its spread. The World Health Organization (WHO) has issued recommendations to help people avoid being infected with the virus. Maintaining a distance of 3 feet between two people and using mask are two safety precautions that people are advised to take.

In large establishments, it is difficult to ensure that people follow these fundamental social distancing norms (Islam et al., 2020a). It is critical to have an automated system in place to easily monitor such violators. The main aim of the system developed is to check whether people in an area maintain social distance among them and also to verify whether they are using mask or not (Bose and Kannan, 2007). The object detection technique is used to detect exactly three classes: masked faces, unmasked faces, and people, to implement the proposed model. The difficulty of face recognition in image processing and computer vision has been quite common. (Goyal et al., 2022) (Karthika and Bose, 2011).

First, real-time video footage of a public place is recorded using a camera. The video recordings are utilized to extract facial images, which are then used to recognize the mask. The Convolutional Neural Network (CNN) learning technique is utilized to extract image features and numerous hidden layers then learn these characteristics. Deep neural networks of the sort known as convolutional neural networks are used most frequently in deep learning (Mani et al., 2022) (Sharon et al., 2022) to interpret visual vision. If the architecture senses individuals without a face mask, the information is forwarded to the relevant authority, who will take the appropriate action. The most effective approaches to stop the spread of illnesses and lessen the impact of the corona virus pandemic when economic activity has resumed are social withdrawal and self-isolation. Many people have been shown to neglect public health programmes, especially when it comes to social distance. This research applies a deep learning model to automatically detect any violation of social distancing protocols in the workplace and public areas. The software uses a video feed to detect and alert people when they are in close proximity to one another. The YOLOv3 approach is employed in an open-source object detection pre-trained model to detect pedestrians through analyzing video frame captures from the camera. Top-down representation of the video frame is built for measuring the distance in 2D plane.

The main objective of this work is to construct a methodology for discovering COVID-19 issues. The violations include face mask violation and social distancing violation. The CCTV cameras shall be used to capture images from the public places, and then these images are fed into the system that determines if the person in the frame are wearing a mask or not and also determines the social distancing measures between the people. The system should keep track of the count of people violating these rules. The authority takes necessary actions based on these information.

The proposed system is composed of two main functionalities. The system tracks and counts the number of individuals wearing face masks and displays the results. The system first differentiates the people wearing and not wearing mask using a CNN model trained on MobileNetV2 architecture. The other functionality is to detect the social distancing violations. The system keeps track of people violating social distancing and the people not violating social distancing. The YOLOv3 architecture, which is trained on the COCO dataset, is utilized by the system to recognize people inside the frame. Subsequently, the Euclidean distance is employed to measure the gap between two people. The remaining portion of the article is divided into various sections to aid in the comprehension of this research work. The previous work used for the detection of face mask using CNN are illustrated in Section 2. The proposed face mask and system for detecting social distance are shown in Section 3. Using a variety of evaluation measures, Section 4 describes and assesses the outcomes of the suggested approach. Section 5, summarizes the findings and offers suggestions for additional enhancement.

## 2 Related work

A CNN-based multi-task learning system was introduced by Lin et al. (Lin et al., 2020) to detect helmet use in tracked motorcycles. This system is able to differentiate between drivers and passengers. The framework for analyzing helmet wear can automatically identify helmet-wearing and helmet-less motorcyclists. Their main objective is to find any motorcycle riders riding without helmets. But their model is not suitable for places with more traffic as they had lesser number of training samples. M. Cristani et al. (Cristani et al., 2020) proposed a Visual Social Distancing Problem to truly detect potentially dangerous situations by calculating the inter-personal distance (the physical distance that individuals choose to maintain between themselves and others 16 while interacting.) from an image while avoiding false alarms.

To identify a person automatically from provided photos, M. S. Ejaz et al. proposed a Convolutional Neural Network (CNN)-based method for Masked face recognition. This model proved to be successful in recognizing faces, whether with or without a mask, and achieved a high recognition accuracy for basic masked faces.W. Bu et al. (Bu et al., 2017) developed a cascading framework for the detection of masked faces and successfully identify possible terrorists’ faces in photos. This paradigm divides people between those who use masks and those who don’t. However, their model over fits as a result of the small number of training samples. A Principal Component Analysis (PCA) implementation was proposed by M. S. Ejaz et al. (Ejaz et al., 2019) for the task of automatically recognizing both masked and non-masked faces from pictures. Deore et al. conducted research on a Masked Face Detection Approach to detect the presence of a masked person in video automatically. This study aimed to identify masked faces in different types of environments. The system was primarily suggested for security reasons. Four distinct steps of masked face detection were examined by their model, along with their effectiveness. A Deep Learning and Machine Learning Based Approach for Person Identification in Group Photos was proposed by A. Sakhapara et al. (Sakhapara et al., 2018) for the purpose of detecting faces in group images and identifying the people present. This approach uses advanced algorithms to recognize and distinguish individuals in a group photo. The system employs two convolutional neural networks to identify people, particularly in a crowd.

Based on a Segmented Region of Interest, Ahamad et al. (2020) suggested a system for tracking the distance between people and generates an alarm when a safety violation occurs. This approach was created to stop the corona virus from spreading by creating a safe distance between individuals in public places. The model recognizes breaches of social distance as well as breaches of accessing forbidden locations, protecting public safety. For the purpose of identifying social distance from video material, Gupta et al. (2020) presented an SD-Measure: A Social Distancing Detector. The techniques employed were Centroid-based Object Tracking and Mask RCNN (Region based Convolutional Neural Network). The framework follows the subjects throughout the video using a centroid tracking technique. Large obstructions in the cameras’ field of vision, however, may interfere with the monitoring of individuals and, as a result, the accurate assessment of social distance. Neelavathy Pari S et al. (Geetha et al., 2020) makes use of mobile camera and bluetooth technology for finding the social distance and the authors have developed an application for smart phone to monitor whether social distancing is maintained. The model tracks social estrangement and notifies smart phone users. Only a little distance can be captured by a smart phone camera. (Sampathkumar et al., 2007; Poongodi et al., 2021a; Poongodi et al., 2021b; Bourouis et al., 2022; Dhiman et al., 2022). The system in (Jignesh Chowdary et al., 2020) automatically identifies presence or absence of a protective mask on the face. It combines the image histograms pixel intensity and visual attributes of CNN. The newest coronavirus (COVID-19) had also significantly proliferated over the globe. While adults are more likely than children to get major infections from coronavirus (COVID-19), children are much more at hazard of COVID-19-related significant sickness and difficulties (Ryumin et al., 2019) (Logeswari et al., 2023) (Wang et al., 2020a). The outcomes of the proposed research showed that the predictive model correctly predicts patients with COVID-19 disease 93% of the time, with recall and precision values of 76.47% and 76.47%, respectively. The investigation demonstrated that the model may help with COVID-19 severity prediction and diagnosis. The study (Maheswaran et al., 2022) recommended the usage of both advanced models such as machine learning and deep learning with the real-time data from the Johns Hopkins dashboard in order to comprehend its normal exponential behavior and the projection of the COVID-2019’s impending accessibility throughout the countries (Wang et al., 2020b) (Li et al., 2020) (Islam et al., 2020b).

In the context of human-machine interaction, the method (Deng et al., 2019) for detecting and recognizing three-dimensional one-handed motions. The system’s module for acquiring a gesture based database are characterized in terms of how conceptually they are grouped together. It has been demonstrated that database consisting of 3D-gesture has a logical structure. The results of automatic face and hand form identification are given. There is insufficient cultural opinion on whether to force people to wear face masks against transmission of corona virus. The researchers (Luo et al., 2022) demonstrates the causes of Severe Acute Respiratory Syndrome Coronavirus 2 (SARS-CoV-2) and analyses the importance of wearing the mask. During pandemic situations, people are advised to use mask and maintain social distancing (Luo et al., 2022). Because of the scarcity of the training samples, most of the above models suffered overfitting problem. To resolve the overfitting problem caused by the low number of training samples, a greater number of complex training samples are used. As a result of this, high accuracy can be obtained.

## 3 Proposed system

**FIGURE 1:**
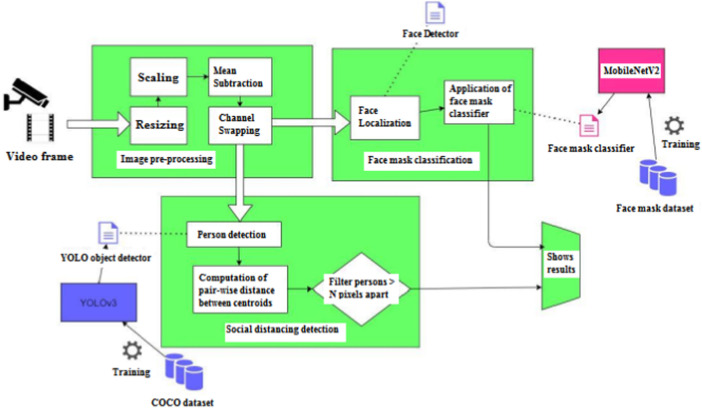
Proposed Architecture of Face mask and Social distancing detection.

The overall framework is presented in Figure 1, emphasizes on the detection of face masks and social distance between people while also increasing detection accuracy.

### 3.1 MobileNetV2

MobileNetV2 is expected to perform more effectively on mobile platforms. It focuses on links between bottleneck levels and an inverted residual structure. The intermediate expansion layer filters non-linearity properties using Light depth-wise convolutions. The architecture of MobileNetV2 consists of a fully convolutional layer with 32 filters and a residual bottleneck of 19 layers as shown in Table 1.

**TABLE 1:** MobilenetV2 architecture.

Input	Operator	t	c	n	s
224*224*3	conv2d	—	32	1	2
112*112*32	bottleneck	1	16	1	1
112*112*16	bottleneck	6	24	2	2
56*56*24	bottleneck	6	32	3	2
28*28*32	bottleneck	6	64	4	2
14*14*64	bottleneck	6	96	3	1
14*14*96	bottleneck	6	160	3	2
7*7*160	bottleneck	6	320	1	1
7*7*320	conv2d 1*1	—	1,280	1	1
7*7*1,280	avgpool7*7	—	—	1	—
1*1*1,280	conv2d 1*1	—	k	—	—

where t represents expansion factor, c denotes number of output channels, n is repeating number, s represents stride.

### 3.2 YOLOv3

**FIGURE 2:**
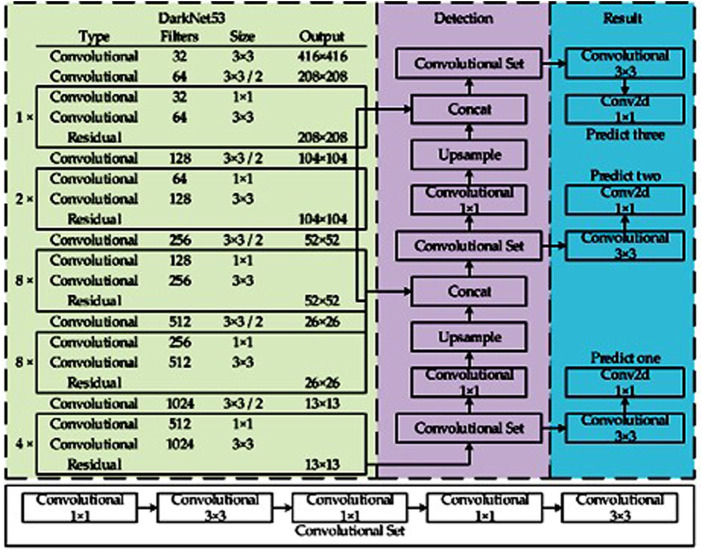
YOLOv3 architecture.

The YOLOv3 object detection approach as shown in Figure 2 is driven by Darknet-53, a convolutional neural network that serves as the backbone. It has 52 convolutions and includes skip connections (similar to ResNet) as well as three prediction heads (similar to FPN), each of which processes the image at a different spatial compression.

Algorithm 1 Face mask classification.

1: **Input: Video frame, Face detector model, Mask detector model**
2: **Output: Centroid coordinates and probability of mask detected**
3:
4: Function detect and predict mask (Frame, face detector model, mask detect or model)
5: *h* ← *height of the frame*
6: *w* ← *width of the frame*
7: *blob* ← *Blob Image*
8: Forward pass the blob through the face detector model
9: **for** *detection* **do**
10:  **if** *confidence* > 0.5 **then** *Get coordinates of the face*
11:  **end if**
12: **end for**
13: **if** *number of faces* > 0 **then** Pass the face ROI through the mask detector model
14: **end if**
15: Return centroid coordinates and corresponding probabilities

Algorithm 2 Pedestrian detector.

1: **Input: Video frame, YOLO detector, personIdx**
2: **Output: Centroid coordinates people in the frame**
3: Function detect_people (Frame, YOLO detector model, personIdx)
4: *h* ← *height of the frame*
5: *w* ← *width of the frame*
6: *blob* ← *Blob Image*
7: Forward pass the blob through the YOLO detector
8: **for** *output layer* **do**
9:  **for** *Detection in output layer* **do**
10:   *scores* ← *probabilities of objects*
11:   *classid* ← *object with maximum probability*
12:   *confidence* ← *scoreofclassID*
13:   **if** *classid* = *personidxand&confidence* > 3 **then** *Append centroid coordinated to centroids* *Append confidence to confidences*
14:   **end if**
15:  **end for**
16: **end for**
17: *idxs* ← *Non*- *Maxima Suppression*(*confidence* = 0.3)
18: **for** *detection in idxs* **do**
19: *Append bounding box coordinates to results*
20: **end for**
21: Return results

Algorithm 3 Business logic.

1: **Input: Video/Live stream of camera**
2: **Output: Video frame with details of people violating face mask and social distancing rules.**
3: *net* ← *YOLO object detector*
4: *faceNet* ← *Face detector model*
5: *maskNet* ← *Mask detector model*
6: ln  ← *Names of output layers from YOLO*
7:
8: **for** *frame* **do**
9:  *frame* ← *read*
10:  *vcount* ← 0
11: *frame* ← *Resize*(*width* = 700)
12: *results* ← *detect people*(*frame*, *net*, ln , *personIdx*)
13: (*locs*, *preds*) ← *detect and predict mask*(*frame*, *facenNet*, *maskNet*)
14:
15: **for** *predictionsin*(*locs*, *preds*): **do**
16:  *color* ← *green*
17:
18:  **if** *Probability*(*without mask*) > *Probability*(*with mask*) **then**
19:   *vcount* ← *vcount* + 1
20:   *color* ← *red*
21: Display bounding box
22:   **end if**
23:  **end for**
24:  **if** *Probability*(*number of predictions in results* > 2) **then**
25:
26:  *prediction* (*x*1, *y*1) *in results*
27:
28: **for** *other prediction* (*x*2, *y*2) *in results* **do**
29: $d\leftarrow \sqrt{{\left(x2-x1\right)}^{2}+{\left(y2-y1\right)}^{2}}$

30:   **if** *d* < *minimum distance* **then** Append the coordinates to serious
31:  **if** *d* > = *minimum distance and&d* < *maximum distanc* **then** Append the coordinates of abnormal
32:   **end if**
33:  **end if**
34:
35: **end for**
36: Display bounding box and violation count=0

### 3.3 Image preprocessing

**FIGURE 3:**
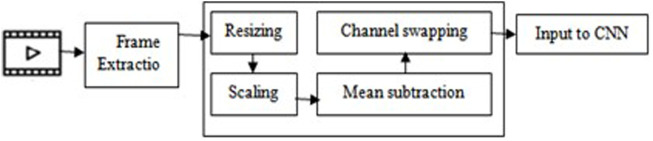
Image preprocessing.

Before moving on to the next phase, the CCTV cameras’ collected images need to be preprocessed. In the preprocessing step, the frames are first extracted from the video or live stream, which are then proceeded to construction of Blob from the image. This is a four-step process which involves resizing, mean subtraction, scaling and channel swapping. The video frame is first resized into 416 × 416 size that the convolution neural network expects. Mean subtraction is then applied to the resized picture. Mean subtraction is used to compensate for variations in lighting in the input pictures. The images are then scaled by a factor of 1/255 and then the channels R and B are swapped since OpenCV assumes images in BGR order. Image preprocessing steps are shown in Figure 3.

### 3.4 Face mask classifier

The COCO dataset is used to train the mask detector. Kaggle is where the dataset was collected. The collection includes 5,000 face-masked photos and 5,000 unmasked images (5,000). This dataset is used by the CNN model to determine whether or not a person is hiding their identity. Preprocessing is first applied to the dataset’s photos. Scaling the pixel intensities in the input range [-1,1], magnifying all the images to 224 × 244 dimensions, and converting to array format are the preparation activities. The preprocessed image and related labels are then added to the lists of data and labels, respectively. The labels are encoded using a one-hot format, where each member of the labels array is made up of an array with only index (1 as the hot index). The data is then divided into 20% for testing and 80% for training using scikit-practical learn’s technique. During training, on-the-fly mutations are applied to the photos in an attempt to enhance generalization. The data augmentation step produces mutations, makes use of the random rotation, zoom, shear, shift, and flip parameters. In the next step, MobileNetV2 is prepared for Fine-tuning. Three steps make up the fine-tuning process:

1. Do not include the network’s head when loading MobileNet with pre-trained ImageNet weights.

2. Create a new FC head, replace the old one, and attach it to the base.

3. Freeze the network’s base layers so that their weights won’t change throughout the back-propagation process while the head layer weights are tweaked.

There are two distinct block types in MobileNetV2. With a stride of 1, the first block is residual. Another is a block with a two-step stride for shrinking. Both kinds of blocks have three layers:

1. A 1 × 1 convolution with ReLU6 makes up the top layer.

2. Convolution in depth makes up the second layer.

3. There is no non-linearity in the third layer, which is another 1 × 1 convolution.

**FIGURE 4:**
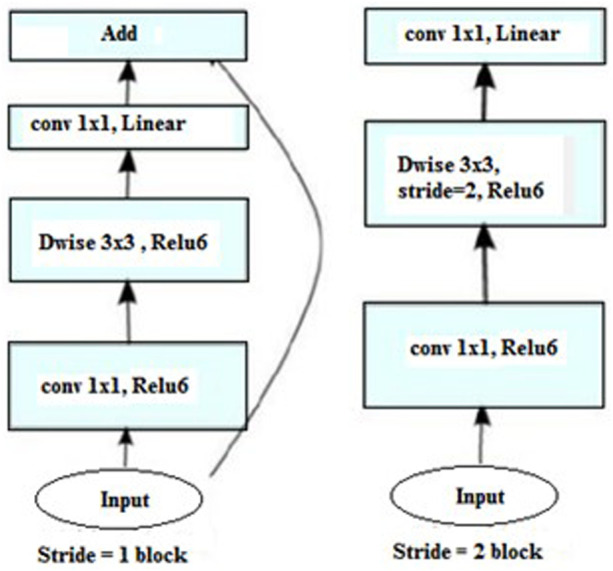
Blocks of MobileNetV2.

In order to predict whether or not a person in an image is using a mask, the trained model can be loaded whenever necessary after being trained over a number of iterations. Figure 4 presents the block of MobileNetV2.

### 3.5 Pedestrian detector

The Blob image from the preprocessing module is subjected to a forward pass through the YOLOv3 network. YOLO is a single stage detector which are faster in performance than R-CNNs. COCO dataset is trained using YOLO. The COCO dataset consists of around 3,30,000 images of 80 classes including People, Bicycle, Cars, Animals, etc. The YOLO makes use of the weights which are used to load the model from disk. After the forward pass through the YOLO network, three outputs each containing the details of bounding boxes, confidence and class IDs is obtained. YOLO does not apply non-maxima suppression, so non-maxima suppression is explicitly applied on the output to ensure any redundant or extraneous bounding boxes are not there. Finally, the obtained probabilities and coordinates is sent for further processing.

### 3.6 Business logic module

The outputs from the face mask detector module and the pedestrian detector module are combined here. Following the completion of the pedestrian detection process, each pedestrian’s centroid is calculated using the obtained bounding box position and a scaling factor to scale the distance. If this distance is greater than some standard threshold value, then the two pedestrians involved are noted to have violated the social distancing norm. The count of people not using masks and not maintaining social distancing at the moment are kept track of and displayed on the screen of the application. This information can then be sent to the proper authority through email if needed to take necessary actions.

### 3.7 Performance enhancement—Multithreading

This is an additional step done to reduce the delay in reading frames from the video/live stream. The idea is to create two threads -t1 (main thread) and t2 (reader thread).

**FIGURE 5:**
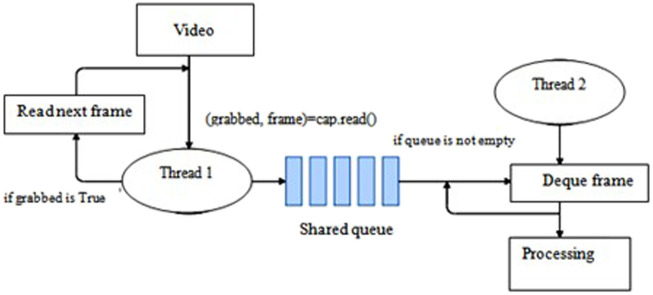
Using multithreading to reduce delay in reading frames.

A shared queue is defined where the reader thread t2 inserts the frame. The main thread, instead of reading the frames directly from the OpenCV buffer, reads the frames from the shared queue. Since the two threads are run simultaneously, the frames are readily available for the main thread to read from the queue, thereby reducing the delay in reading the frames Figure 5 using multithreading to reduce frame size.

## 4 Results and discussion

**FIGURE 6:**
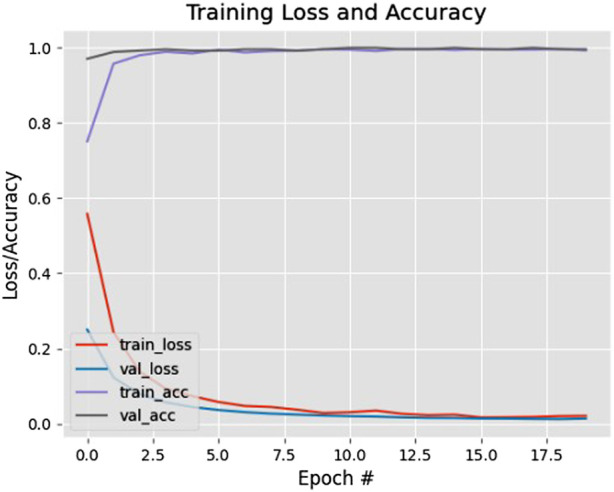
Accuracy/Loss plot of Face mask classifier.

The proposed system makes advantage of the YOLOv3 architecture to identify pedestrians. MobileNetV2 architecture is used to train the system. TensorFlow and OpenCV are utilized to implement the proposed method. To obtain the final output, the model is fed the video input’s frames. The graph in Figure 6 shows the accuracy/loss curves obtained by the training of the face mask classifier.

**FIGURE 7:**
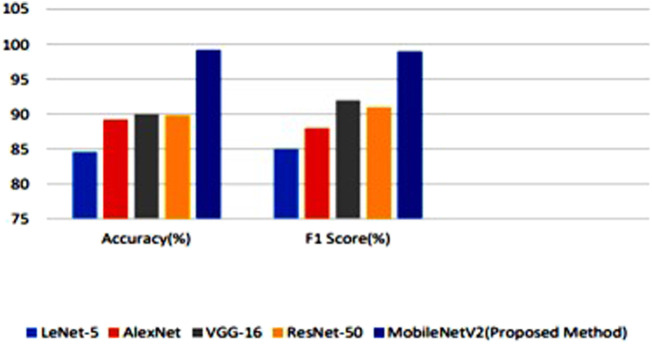
Comparative Analysis of proposed Face Mask detection system with various models.

The MobileNetV2 model which was used for face mask detection was compared with various existing systems. Figure 7 demonstrates the comparison results of different detection model.

**FIGURE 8:**
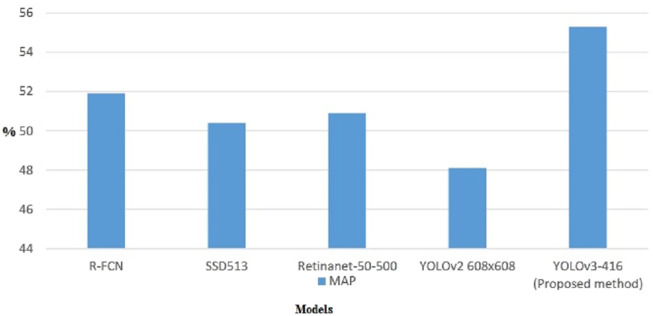
YOLOv3 vs. Other object detectors on COCO dataset—mAP.

The newly trained YOLOv3 is also trained with other deep learning models. Figure 8 presents the comparison results of various existing techniques with the proposed system.

## 5 Conclusion and future work

Given the COVID-19 crisis, using a mask could become compulsory in future. The proposed model would make a significant contribution to the public healthcare system. Utilizing basic machine learning algorithms and streamlined methodologies, the suggested strategy has produced results with a respectable level of accuracy. The model could be improved to determine whether or not the mask is surgical, N95, or otherwise susceptible to viruses. Social distance is one of the most efficient measures to reduce physical contact that can cause the corona virus to spread. A significant degree of accuracy was achieved in the proposed model’s identification of social distancing. The results, however, demonstrate that individuals are difficult to accurately discover in outdoor settings and challenging scenarios with distant sights using the object detection framework that was employed for that purpose. The potential applications of the proposed solution in other areas beyond smart healthcare systems is public transportation or public gatherings, where face mask detection is becoming increasingly important.For future development, a more robust object detection model could be used. The performance of the model can be enhanced incorporating additional information such as facial expressions and head poses.

## Data Availability

The original contributions presented in the study are included in the article/supplementary material, further inquiries can be directed to the corresponding author.

## References

[B1] Ahamad A. H. Zaini N. Latip M. F. A. (2020). “Person detection for social distancing and safety violation alert based on segmented roi,” in 2020 10th IEEE international conference on control system, computing and engineering (ICCSCE) (IEEE), 113–118.

[B2] Bose S. Kannan A. (2007). Adaptive multipath multimedia streaming architecture for mobile networks with proactive buffering using mobile proxies. J. Comput. Inf. Technol. 15, 215–226. 10.2498/cit.1000884

[B3] Bourouis S. Band S. S. Mosavi A. Agrawal S. Hamdi M. (2022). Meta-heuristic algorithm-tuned neural network for breast cancer diagnosis using ultrasound images. Front. Oncol. 12, 834028. 10.3389/fonc.2022.834028 35769710 PMC9234296

[B4] Bu W. Xiao J. Zhou C. Yang M. Peng C. (2017). “A cascade framework for masked face detection,” in 2017 IEEE International Conference on Cybernetics and Intelligent Systems (CIS) and IEEE Conference on Robotics, Automation and Mechatronics (RAM) (IEEE), 458–462.

[B5] Cristani M. Del Bue A. Murino V. Setti F. Vinciarelli A. (2020). The visual social distancing problem. Ieee Access 8, 126876–126886. 10.1109/access.2020.3008370

[B6] Deng J. Guo J. Xue N. Zafeiriou S. (2019). “Arcface: Additive angular margin loss for deep face recognition,” in Proceedings of the IEEE/CVF conference on computer vision and pattern recognition, 4690–4699. 10.1109/TPAMI.2021.3087709 34106845

[B7] Dhiman P. Kukreja V. Manoharan P. Kaur A. Kamruzzaman M. Dhaou I. B. (2022). A novel deep learning model for detection of severity level of the disease in citrus fruits. Electronics 11, 495. 10.3390/electronics11030495

[B8] Ejaz M. S. Islam M. R. Sifatullah M. Sarker A. (2019). “Implementation of principal component analysis on masked and non-masked face recognition,” in 2019 1st international conference on advances in science, engineering and robotics technology (ICASERT) (IEEE), 1–5.

[B9] Geetha A. Vasu B. Jeevitha V. (2020). Monitoring social distancing by smart phone app in the effect of covid-19.

[B10] Goyal H. Sidana K. Singh C. Jain A. Jindal S. (2022). A real time face mask detection system using convolutional neural network. Multimedia Tools Appl. 81, 14999–15015. 10.1007/s11042-022-12166-x PMC8874748 35233179

[B11] Gupta S. Kapil R. Kanahasabai G. Joshi S. S. Joshi A. S. (2020). “Sd-measure: A social distancing detector,” in 2020 12th International conference on computational intelligence and communication networks (CICN) (IEEE), 306–311.

[B12] Islam M. S. Moon E. H. Shaikat M. A. Alam M. J. (2020). “A novel approach to detect face mask using cnn,” in 2020 3rd International Conference on Intelligent Sustainable Systems (ICISS) (IEEE), 800–806.

[B13] Islam M. S. Rahman K. M. Sun Y. Qureshi M. O. Abdi I. Chughtai A. A. (2020). Current knowledge of Covid-19 and infection prevention and control strategies in healthcare settings: A global analysis. Infect. Control Hosp. Epidemiol. 41, 1196–1206. 10.1017/ice.2020.237 32408911 PMC7253768

[B14] Jignesh Chowdary G. Punn N. S. Sonbhadra S. K. Agarwal S. (2020). “Face mask detection using transfer learning of inceptionv3,” in International Conference on Big Data Analytics (Springer, 81–90.

[B15] Karthika S. Bose S. (2011). A comparative study of social networking approaches in identifying the covert nodes. Int. J. Web Serv. Comput. (IJWSC) 2, 65–78. 10.5121/ijwsc.2011.2306

[B16] Li T. Liu Y. Li M. Qian X. Dai S. Y. (2020). Mask or no mask for Covid-19: A public health and market study. PloS one 15 15, e0237691. 10.1371/journal.pone.0237691 PMC7428176 32797067

[B17] Lin H. Deng J. D. Albers D. Siebert F. W. (2020). Helmet use detection of tracked motorcycles using cnn-based multi-task learning. IEEE Access 8, 162073–162084. 10.1109/access.2020.3021357

[B18] Logeswari G. Bose S. Anitha T. (2023). An intrusion detection system for sdn using machine learning. Intelligent Automation Soft Comput. 35 35, 867–880. 10.32604/iasc.2023.026769

[B19] Luo S. Li X. Zhang X. (2022). “Wide aspect ratio matching for robust face detection,” in Multimedia tools and applications, 1–18. 10.1007/s11042-022-13667-5 PMC9444702 36090154

[B20] Maheswaran N. Bose S. Logeswari G. Anitha T. (2022). “Multistage intrusion detection system using machine learning algorithm,” in Mobile computing and sustainable informatics (Springer, 139–153.

[B21] Mani S. Sundan B. Thangasamy A. Govindaraj L. (2022). “A new intrusion detection and prevention system using a hybrid deep neural network in cloud environment,” in Computer networks, big data and IoT Springer, 981–994.

[B22] Poongodi M. Malviya M. Hamdi M. Rauf H. T. Kadry S. Thinnukool O. (2021). The recent technologies to curb the second-wave of Covid-19 pandemic. Ieee Access 9, 97906–97928. 10.1109/ACCESS.2021.3094400 34812400 PMC8545196

[B23] Poongodi M. Sharma A. Hamdi M. Maode M. Chilamkurti N. (2021). Smart healthcare in smart cities: Wireless patient monitoring system using iot. J. Supercomput. 77, 12230–12255. 10.1007/s11227-021-03765-w

[B24] Ryumin D. Kagirov I. Ivanko D. Axyonov A. Karpov A. (2019). “Automatic detection and recognition of 3d manual gestures for human-machine interaction,” in International archives of the photogrammetry, remote sensing & spatial information sciences.

[B25] Sakhapara A. Pawade D. Dedhia S. Bhanushali T. Doshi V. (2018). “Machine learning based approach for person identification in group photos,” in 2018 Fourth International Conference on Computing Communication Control and Automation (ICCUBEA) (IEEE), 1–5.

[B26] Sampathkumar V. Bose S. Anand K. Kannan A. (2007). “An intelligent agent based approach for intrusion detection and prevention in adhoc networks,” in 2007 International Conference on Signal Processing, Communications and Networking (IEEE), 534–536.

[B27] Sharon A. Mohanraj P. Abraham T. E. Sundan B. Thangasamy A. (2022). “An intelligent intrusion detection system using hybrid deep learning approaches in cloud environment,” in International Conference on Computer, Communication, and Signal Processing (Springer, 281–298.

[B28] Wang J. Pan L. Tang S. Ji J. S. Shi X. (2020). Mask use during Covid-19: A risk adjusted strategy. Environ. Pollut. 266, 115099. 10.1016/j.envpol.2020.115099 32623270 PMC7314683

[B29] Wang Z. Wang G. Huang B. Xiong Z. Hong Q. Wu H. (2020). Masked face recognition dataset and application. arXiv preprint arXiv:2003.09093.

